# Reasons for admission and rehabilitation rates of various wildlife species in Finland

**DOI:** 10.3389/fvets.2024.1455632

**Published:** 2024-10-02

**Authors:** Kati White, Laura Hänninen, Sanna Sainmaa, Anna Valros

**Affiliations:** ^1^Faculty of Veterinary Medicine, Research Centre for Animal Welfare, Helsinki University, Helsinki, Finland; ^2^SEY, Animal Welfare Finland, Helsinki, Finland; ^3^Helsinki Zoo, Helsinki, Finland

**Keywords:** wildlife, rehabilitation, species, hedgehog, release rates

## Abstract

Wildlife rehabilitation is a common part of animal-protection work. In Finland wildlife care is usually based on volunteer work and no licensing or training is required. Wildlife casualties are also treated professionally in some contexts such as zoos. The species of wildlife casualties may influence treatment decisions. Our anonymous online survey examined wildlife caregiving practices in Finland (*n* = 78), focusing on the care provided to various animal species and the outcomes of rehabilitation efforts. The survey was sent to both veterinarians and volunteers caring for wildlife, and it was part of a larger survey. Questions were mainly closed, and opinion-related questions were applied on a Likert scale (1–7; where 1 meant strongly disagree and 7 meant strongly agree). Most respondents primarily cared for mammals and birds. Reptiles, amphibians, and fish received less attention. Injuries and overwinter survival, especially in the case of hedgehogs, were the primary reasons for wildlife admissions. The training background of the rehabilitators varied and was related to the animal species being treated. Those caring mainly for hedgehogs (*Erinaceus europaeus*) were the least likely to have animal-related training or long-term experience in wildlife care. We show a notably high rehabilitation rate of approximately 80% of commonly treated species, significantly surpassing figures from other countries, which raises concerns that animals are admitted or released on too light grounds, leading to animal welfare problems. It is also noteworthy that only one-fifth of respondents said they kept records of animal admissions. Less than 40% of respondents emphasized the need for further education on any specific issue, which may indicate overestimation of personal skills. In conclusion, our study raises concerns regarding the ethics and potential harm associated with wildlife rehabilitation.

## 1 Introduction

In Finland, wildlife care is usually based on volunteer work by non-governmental organizations (NGOs) and citizens, although wildlife casualties are treated professionally in some contexts such as zoos. Unlike in some other countries (1–3), no licensing or specific training is required for treating wildlife in Finland, and to date no centralized statistics have been collected on wildlife casualties. The new Animal Welfare Act in Finland (4) requires that, from the beginning of 2024, wildlife rehabilitators report their activities to the authorities and keep records of the animals they treat. According to the same law, care measures other than providing first aid are illegal without notifying the authorities. The animal should then be either delivered to a registered wildlife care facility, released, or euthanized (4).

Practices and principles of care may vary significantly between wildlife rehabilitators, for example which species are cared for, how records are kept, the rehabilitation process, and the reasons for performing euthanasia (2, 5). Person’s background education may also affect their reasoning when rehabilitating or euthanizing wildlife casualties under their care (6). Veterinarians treating wildlife in Finland were overall more willing than wildlife rehabilitators with other training to euthanize a wildlife casualty and stressed the importance of returning an injured animal back into the wild after treatment. In contrast, those rehabilitators with no formal animal-related training in Finland placed more emphasis on the continuation of an animal’s life, albeit dependent on humans and even if causing the animal a great deal of stress. They were also overall the least willing to use euthanasia (6). Similar attitudes have also been shown elsewhere (2, 7).

Treatment reasons and the likelihood of recovery and survival after release can vary considerably between wildlife species, and these reasons should also influence decision-making (5, 8, 9). Further, as not everyone has the capacity to care for species with more demanding management requirements, we assume that various management and rehabilitation requirements between species also affect the treatment decisions. Species are also more likely to be managed if the public encounters them more often, if they are perceived positively, and if the animals are easy to catch. For example, the number of hedgehogs (*Erinaceus europaeus*) brought into care is increasing in many countries (10, 11). Hedgehogs are easy to catch, and care tips are shared in social media hedgehog care groups. Wild animals may thus be cared for by people who have no animal-related formal training, which may influence their care decisions (6). However, not many studies have investigated the association between the species that rehabilitators mainly care for and their treatment decisions and outcomes.

Whether an animal can be returned to the wild at all or whether its future life is dependent on humans is also a question (6). More discretion should be exercised when admitting wild animals into care than for domestic species. Care is inherently stressful for wild animals and may also lead to physical harm or even death, and unnecessary care should therefore be avoided (12–14). Assessments and decisions of possible treatments, including euthanasia, should be made as soon as possible when a wildlife casualty is presented, not only to prevent suffering of the animal but also to ensure staff safety (8, 15).

When a wildlife casualty is admitted into care, not all animals survive until they are ready to be released back into the wild. Also, not all individuals that survive can be released. Rehabilitation rates are commonly used as a measure of care success; however, whether this is a good measure is debatable (2, 5, 9). Release rates vary greatly between rehabilitators for several reasons, such as record-keeping methods including whether for example animals euthanized on arrival are recorded at all, standard of care, euthanasia rates, standards for evaluating whether an animal is fit for release, and the state of the animals at admission (2, 5). Both very high and low release rates may be indicative of poor wildlife management processes, resulting in poor animal welfare (5).

We have previously reported on the overall agreement among persons treating injured wildlife on euthanasia-related questions and on the effects of background information (6). Here, we aimed to analyze whether differences in opinions and practices occur that can be linked to the wildlife species that the respondent mainly cares for. We also wanted to see which species are admitted, for what reasons, what the outcomes are, whether rehabilitators caring for different species show any background differences, and what further training the rehabilitators themselves consider they need. This information is relevant for gaining more knowledge of the current situation regarding wildlife care and for investigating whether possible animal welfare indicators, such as very high or low release rates, can be identified.

## 2 Materials and methods

In spring 2020, we posted a web-based, anonymous questionnaire (Qualtrics^xm^, Seattle, United States) aimed at Finnish Facebook groups for veterinarians and volunteers caring for wildlife. These groups included the member associations of SEY Animal Welfare Finland, Eläinten pelastusrinki (Animal Rescue Circle), a Facebook-site for hedgehog rehabilitators, Animal Welfare Advisors of SEY Animal Welfare Finland and a veterinarian’s Facebook community. The questionnaire was open for approximately 1 month. Rehabilitators may also have spread the questionnaire to other Facebook groups that we do not know of. Information about the questionnaire was further spread through the Animal Welfare Research Center’s Facebook-site and by directly e-mailing professional rehabilitators at two zoos, volunteer rehabilitators practicing within the largest Finnish animal welfare organization, SEY Animal Welfare Finland, The Finnish association for Nature Conservation and bird conservation associations. SEY’s volunteers were also reached through a newsletter.

This questionnaire was part of a larger one comprising of 12 sections with questions concerning euthanasia and wildlife and admitting wild animals in for treatment and rehabilitation. Questions were mainly closed, and opinion-related questions were applied on a Likert scale (1–7; where 1 meant strongly disagree and 7 meant strongly agree). The content is explained in more detail in White et al. (6). The questions of which results are reported here are listed in Supplementary material S1.

In the absence of a readily available questionnaire, we formulated the items based on our aims. Before launching the questionnaire, we sent it for feedback to four non-veterinarians and four veterinarians caring for wildlife casualties to make sure that the content was appropriate, and the questionnaire was slightly modified accordingly.

Here we report results on the species mainly being cared for; reasons why animals are brought to respondents, numbers of animals that are rehabilitated back into the wild or for which other solutions are taken, record keeping (i.e., whether answers were based entirely or partly on respondents’ personal records or on their own assessment), and whether experience with different species affects how respondents perceive euthanasia and rehabilitation choices. We also asked respondents to name a typical individual wildlife patient brought to their care and to explain the typical end-of-life solution that is usually used on the given species. In addition, we analyzed the most-needed further education that respondents stated in the survey.

The larger questionnaire also included a set of items on background factors. Of these, we analyzed the differences in animal-related education between respondents caring for different species. To make sure the differences are not due to other demographic factors, we also included information on gender, age (birth year), experience of treating wild animals in years, and individuals treated per year. We included all respondents who had answered the three sections of questions analyzed here. Overall, they had answered at least 95% of the questions included in the whole survey. All respondents had a recent history of caring for wildlife casualties.

Responses were anonymous. We followed the guidelines of the Finnish National Board on Research Integrity (TENK) (16), according to which no ethical review was required.

### 2.1 Statistics

Before conducting the statistical analyses, we classified each respondent’s animal-related education as veterinarian, other (such as a trained rehabilitator, biologist, or veterinary nurse), and none. We further classified the animals being cared for into three categories: mammals, birds, and amphibians, reptiles, and fish (which were merged into one category). Mammals and birds were further classified into the following species groups based on whether they were cared for: Mammals as (1) respondent did not care for any mammals, (2) respondent indicated that the species most commonly cared for was only hedgehogs or (3) respondent cared for all mammals, and birds as (1) respondent did not care for any birds, (2) respondent cared for large and predatory bird species, or (3) respondent cared for other than large bird species or birds of prey. The example species for which respondents estimated the treatment outcomes were classified as birds or mammals, as six respondents used the unclassified generic terms “bird” that prevented a more precise bird species classification. Finally, the example mammals were further classified as hedgehogs and other mammals. Hedgehogs were the most common species reported to be cared for solely.

Because the data did not follow a normal distribution, we used Kruskal-Wallis tests to assess the differences between the educational groups, their ages, work experience with admitted species, admission reasons, and numbers of animals being cared for. Differences in treatment outcomes were examined both by comparing differences between birds and mammals using the Mann–Whitney U test and between respondent categories based on the species group that respondents primarily cared for using the Mann–Whitney U test (for amphibians, reptiles, and fish) or the Kruskal-Wallis test (for others). Kruskall-Wallis and Mann–Whitney U tests were also used for testing differences between perceptions of euthanasia and rehabilitation between respondents caring for different species. These comparisons were made within each animal category only, due to some persons caring for several species categories. We used *χ*^2^-tests to identify differences in animal-related background education between respondents caring for different species and differences in further education requirements. Pairwise comparisons were Bonferroni-corrected.

We used IBM SPSS Statistics version 27 (IBM Inc. Chicago, IL) for analyzing the data. Significance was declared at *p* ≤ 0.05.

## 3 Results

### 3.1 Overall

Of the 119 respondents, 78 had answered enough questions (at least 95%) to be included in the analysis. Respondents’ median (min–max) age was 43.5 (23–75) years, and they had been caring for wildlife casualties for an average of 5 (0–70) years. Most reported being female (91% female, 4.5% male, and 1.3% other).

**Figure 1:**
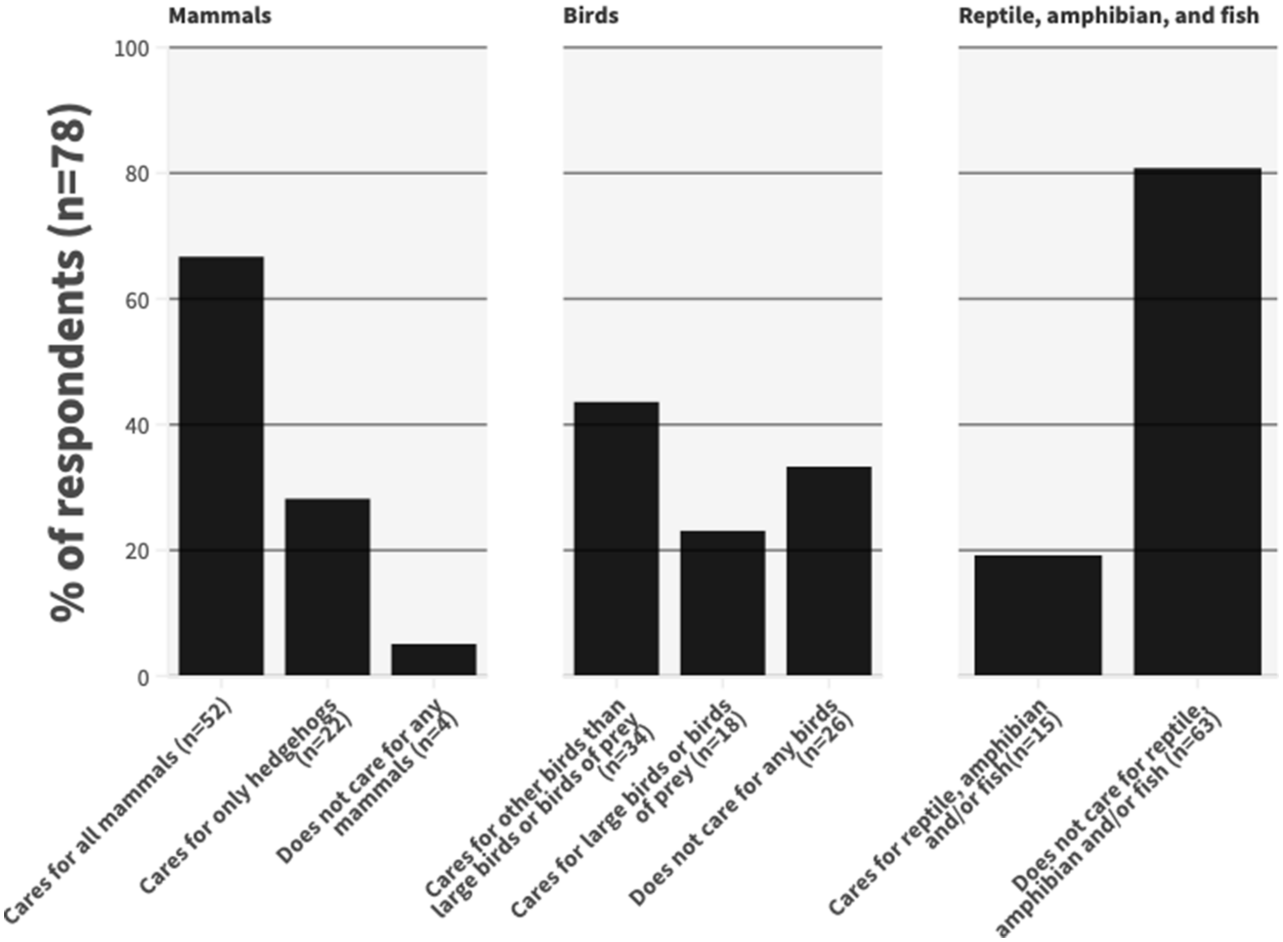
Categories of wildlife species that respondents (*n* = 78) reported caring for.

Based on the responses, more than two thirds of respondents managed all mammal species and almost a third said they only managed hedgehogs. Of all respondents, less than a quarter said they mainly managed large birds and birds of prey, and more than two quarters managed other species. The mammal and bird species that respondents most commonly reported caring for are listed in Table 1. Reptiles were managed by less than a fifth of respondents (Figure 1). The species categories overlapped, as 16.7% (13/78) reported caring for wildlife casualties from all species categories, and 44.9% (35/78) reported caring for individuals from two species categories, mostly birds and mammals (97.1%, 34/35).

**Table 1:** The species that respondents reported most commonly caring for (they could list one or more species).

Species	Proportion (*n*) of all 78 respondents^a^
Hedgehogs	80% (62)
Squirrel	36% (28)
Hares and rabbits	17% (13)
Crows	17% (13)
Small birds (other than swifts)	15% (12)
Seagulls	14% (11)
Common swift	13% (10)
Swans	12% (9)
Owls	8% (6)
Common lizard	8% (6)
Pigeons	6% (5)
Deer	5% (4)
Hawks	5% (4)
Canines	4% (3)
Ducks	4% (3)
Trushes	4% (3)
Toads	4% (3)
Grass snakes	4% (2)
Vipers	3% (2)
Brown frogs	3% (2)
Common newts	3% (2)

a In addition, the following species were mentioned once; otters, seals, voles, moles and mice, common teals, white-cheeked goose, waxwings, toads, eastern slow worms, perch.

Respondents reported having cared for a median (interquartile range; IQR) of 15 (59) individual wildlife casualties per year. This number varied between the species subcategories (*p* < 0.05 for all): those who cared for all mammals cared for a larger number of animals each year than those who cared for only hedgehogs or did not care for any mammals at all. Those who cared for birds, reptiles, amphibians, and fish cared for more animals per year than those who did not care for these species at all (Table 2).

**Table 2:** Median (interquartile range) age, years of work experience, and number of wildlife casualties cared for annually, by subclasses.

Species categories and *p*-values	Subcategories within species cared for by respondents	Background factors		
		Age	Length of experience	No. of treated animals per year
Mammals	None (*n* = 4)	51 (37)a	**2 (5)ab**	**22 (61)ab**
	Only hedgehogs (*n* = 22)	46 (24)a	**2.5 (5)a**	**5 (16)a**
	All mammals (*n* = 52)	43 (15)a	**7 (12)b**	**28 (85)b**
*p* (df = 2)		ns	**0.02**	**0.003**
H(*n* = 78)		2.90	**7.52**	**11.58**
Birds	None (*n* = 26)	44 (21)a	2.5 (7)a	**5 (16)a**
	Large and predatory birds (*n* = 18)	49 (10)a	9(12a)	**27 (58)b**
	Other birds (*n* = 34)	42 (17)a	5 (10)a	**30 (90)b**
*p* (df = 2)		ns	ns	**0.001**
H (*n* = 78)		5.38	4.87	**13.04**
Amphibians, reptiles, and fish	None (*n* = 63)	43 (18)a	**4 (9)a**	**10 (37)a**
	Care for (*n* = 15)	44 (17)a	**12 (27)b**	**60 (197)b**
*p* (df = 1)		ns	**0.003**	**0.003**
z (*n* = 78)		−0.22	**−2.95**	**−2.98**

Different letters within a column mark a difference between the animal categories and species groups.Columns with statistically significant results are in bold.

No differences were found in respondent ages between those caring for different wildlife species, but some differences were found in the work experience of respondents caring for mammals or for amphibians, reptiles, and fish (*p* < 0.05 for both): those who only cared for hedgehogs had a smaller median for work experience years than those who cared for all mammals, and those who cared for amphibians, reptiles, and fish had a larger median for work experience years than those who did not care for these species at all (Table 2).

Most veterinarians (92.9%, 13/14) and respondents with other animal-related education (93.3%, 15/16) responded caring for all mammals, as did 50% of respondents with no animal-related education (24/48). Animal-related background education differed between persons caring for birds and mammals (*χ*^2^ (4, *n* = 77) = 19.92, *p* = 0.001 and *χ*^2^ (4, *n* = 78) = 17.05, *p* = 0.002, respectively). Animal-related education was rare among respondents caring only for hedgehogs (*p* = 0.002); 95.5% (21/22) had no animal-related education while 4.5% (1/22) had other than veterinary education. On the other hand, animal-related education was common among respondents treating large and predatory birds (*p* < 0.001), as only 10.4% (5/48) had no animal-related training, but 50% (7/14) and 40% (6/15) had either veterinary or other animal-related education, respectively (*p* < 0.05 for both).

### 3.2 Reasons for care

Injury (80.8%, *n* = 63) and concern that the animal will not survive the winter (overwinter survival) (67.9%, *n* = 53) were the most common reasons given by respondents for admitting a wild animal into care (multiple reasons could be chosen), an equal proportion of admitted animals were assumed orphans or sick (59%, *n* = 46 for both), and abnormal behaviors were the least common reason for admission (34.6%, *n* = 27).

Reasons for being admitted into care differed somewhat between mammal species; hedgehogs were admitted more often for overwinter survival than other mammals were, and injuries and illnesses were more common admission reasons for other mammals than hedgehogs. Injuries were also more often a reason to admit large and predatory birds into care than other birds (*p* < 0.05 for all). No other statistically significant differences were observed within species classifications (Table 3).

**Table 3:** The median (interquartile range) proportion of admission reasons for an individual animal by subcategories of wildlife casualty species cared for.

Admitted species and *p*-values		Reasons for admission				
		Injury	Overwinter survival	Assumed orphan	Sick	Abnormal behavior
Mammals	None (*n* = 4)	**100%(4)ab**	**0%(0)a**	50%(2)a	**50%(2)ab**	25%(1)a
	Only hedgehogs (*n* = 22)	**50%(11)b**	**96%(21)b**	46%(10)a	**36%(8)b**	36%(8)a
	All mammals (*n* = 52)	**92%(48)a**	**64%(33)c**	67%(35)	**69%(36)a**	35%(18)a
*p* (df = 2)		**<0.0001**	**0.03**	ns	**0.03**	ns
*χ*^2^ (*N* = 78)		**18.82**	**16.92**	3.27	**7.04**	0.19
Birds	None (*n* = 26)	**58%(15)a**	**92%(24)a**	54%(14)a	46%(12)a	42%(11)a
	Large and predatory birds (*n* = 18)	**89%(16)ab**	**57%(10)b**	57%(10)a	72%(13)a	28%(5)a
	Other birds (*n* = 34)	**94%(31)b**	**58%(19)b**	67%(22)a	64%(21)a	33%(11)a
*p* (df = 2)		**0.001**	**0.006**	ns	ns	ns
*χ*^2^ (*N* = 78)		**13.23**	**10.11**	1.17	3.37	1.06
Reptile, amphibian, and fish	None (*n* = 63)	78%(49)a	**43%(40)a**	56%(35)a	57%(36)a	30%(19)a
	Care for (*n* = 15)	93%(13)a	**93%(13)b**	79%(11)a	71%(10)	57%(8)a
*p* (df = 1)		ns	**0.03**	ns	ns	ns
z (*N* = 78)		1.67	**4.60**	2.52	0.97	3.66

Letters on a row mark differences in the admission reasons within species.Columns with statistically significant results are in bold.

Some differences were found between respondents from different backgrounds in the proportions of admitted wild animals for different reasons. Admission into care for overwinter survival was the least common reason among veterinarians, which differed from respondents with no animal-related training (29%, 4/14 vs. 83%, 40/48, *χ*^2^ (2, *N* = 78) = 15.69, *p* = 0.001). Respondents with other animal-related training did not differ from the other two groups (63%, 10/16). Presumably orphaned wild animals were also admitted less frequently by veterinarian respondents than by respondents with other animal-related training (35.7%, 5/14 vs. 81.3%, 13/16, (*χ*^2^ (2, *N* = 78) = 6.47, *p* = 0.04)). Respondents with no animal-related training did not differ from either of the other two groups (60.4%, 29/48). No other differences were observed between respondents with different background education.

### 3.3 Treatment outcomes

The respondents were asked to consider the treatment outcomes through one species they commonly cared for. Of the respondents 58 reported outcomes of a mammal species and out of these 36 were hedgehogs. Other mammals mentioned were squirrels (*n* = 10), foxes (2) hares (1) and voles (1). Twenty-one respondents reported outcomes of birds, and most commonly reported species or groups were swans (*n* = 3), owls (*n* = 3) and seagulls (*n* = 2). Overall, respondents estimated that a median (IQR) of 80% (35) of treated animals were returned to the wild, 4% (10) were euthanized during treatment, 2% (6) died during treatment, and 5% (10) were euthanized before treatment. Very few, 0% (0) were placed in zoos, with no difference in record types, species category, or background training.

Of these figures, 21% were based on respondents’ personal records, 31% on their own assessments, and 49% on both. However, the reported treatment outcomes differed by record type for the proportions of wildlife casualties euthanized before care or casualties died during care (H(2) = 9.20 and H(2) = 7.98, *p* < 0.05, respectively, *n* = 78 for both): Respondents who based their reported figures solely on their own estimates reported a higher median (IQR) proportion of animals euthanized before being admitted into care than did respondents whose figures were based wholly or partly on their own record keeping; 13% (29) vs. 0% (10) and 1% (5), respectively (*p* < 0.05 for both). On the other hand, however, respondents who based their reported figures solely on their own estimates reported that a lower proportion of animals died during care compared to those who based their figures wholly or partly on their personal record keeping; 0€ (4) vs. 3% (8.5) (*p* = 0.03).

Some differences were observed between the treatment results of treated birds and mammals. A lower proportion of birds than mammals were returned to the wild after treatment; 75% (30) vs. 90% (34) (*p* = 0.006, z = −2.74, *n* = 78), and a correspondingly higher proportion of birds than mammals tended to be euthanized before or during treatment (7.5% (18.8) vs. 2% (10), z = −1.95 or 10% (20) vs. 1% (9), z = −1.96, respectively, *p* = 0.05, *n* = 78 for both). However, we also observed differences within the mammal group, with a lower median proportion of hedgehogs than other mammals euthanized during treatment (2% (8) vs. 8% (13), z = −2.26, *p* = 0.02, *n* = 78). No other differences in treatment outcomes were observed.

The proportion of animals that respondents reported returning to the wild or euthanizing prior to treatment differed by respondent background education (H(2) = 16.53 or H(2) = 18.97, respectively, *p* < 0.001 and *N* = 78 for both): veterinarians estimated that a lower median (IQR) proportion of wild animals were returned to the wild than did respondents with other animal-related education or no animal-related education; 50% (19) vs. 80% (29) or 90% (19) (*p* < 0.05 for both). Veterinarians also estimated that a higher proportion of animals had been euthanized before treatment than did respondents with no animal-related training: 40% (45) vs. 0% (5) (*p* < 0.001). Respondents with other animal-related training did not differ from either with 5 (16)%.

The annual number of animals cared for did not associate with the proportion of animals returned to the wild. However, it positively associated with the proportion of animals euthanized during or after care and with dying during care (Spearman rank Corr 0.34, 0.32, and 0.34, respectively, *p* < 0.01, *n* = 78).

### 3.4 Respondents’ perceptions of reasons leading to either euthanasia or rehabilitation

We identified differences in a few questions regarding rehabilitation and euthanasia based on which animal species respondents stated caring for (*p* < 0.05 for all). Respondents who cared for hedgehogs agreed the most with the statement “*I think a wild animal should be rehabilitated if there is even a small chance that the animal can be returned to the wild*” than did respondents caring for all mammals, while respondents treating no mammals did not differ from either of the other two groups; 7(2) vs. 4.5(4) and 7(3), respectively *p* = 0.03, *χ*^2^(2) = 6.83. Respondents treating amphibians, fish, and reptiles agreed less with the statement “*I think a wild animal should be rehabilitated if it is quite likely that the animal can be returned to the wild*” than respondents not treating amphibians, reptiles, or fish; 5 (1) vs. 6 (1), *p* < 0.05, z = −2, respectively.

The stress of treatment experienced by birds and the suspicion of contagious diseases influenced respondents caring for large birds and birds of prey; they were more likely to choose euthanasia than were respondents caring for other birds or not caring for birds at all. Those respondents caring for large birds and birds of prey agreed more with the statements “*I end up with euthanasia if I evaluate that the treatment will cause the animal a lot of stress*”, than respondents not caring for birds at all; 5(2) vs. 3.5(3). Respondents treating other birds did not differ from either of the two groups; 4(2), *χ*^2^(2) = 6.32, *p* = 0.04. The respondents caring for large birds and birds of prey also agreed more with “*I end up with euthanasia if the animal may spread contagious diseases to the wild,”* which differed from respondents not treating birds at all and those treating other birds; 6(3) vs. 4(3) and 4(3), *χ*^2^(2) = 9.04, respectively, *p* = 0.001. Further, respondents treating reptiles, amphibians, and fish agreed more with the statement “*I end up euthanizing animals brought to my care because there is no veterinary help available for them”* than did respondents not treating such species; 4.5(5) vs. 2.5(4), z = −2, *p* < 0.05. Please see overall results in our previous article (6).

### 3.5 Need for further education

Overall, 39.7% (31/78) of respondents felt they needed more information on the provision of medical interventions, while 38.5% wanted additional information on both prognosis assessment and the provision of an appropriate diet (30/78 both), 35.9% (28/78) on assessing the need for care, and 30.8% (24/78) on the provision of non-medical care. In addition, 29.5% of respondents felt that they mostly needed more skills to both assess the condition of an animal to be released into the wild and to take practical steps for returning the animal to the wild (23/78 both), 23.1% (18/78) needed more information to assess the ethical benefits and harms of treatment, and 17.9% (14/78) needed more information to teach young animal’s species-specific behaviors needed to survive in the wild. No differences were found between basic animal-related training, the species cared for, or caring experience in terms of the additional information needs reported.

## 4 Discussion

Most respondents who cared for wildlife casualties in Finland cared for mammals and birds. Reptiles, amphibians, and fish were cared for by less than one-fifth of the respondents, who cited a lack of qualified veterinarians as one reason for this low value. The most common reasons for caring for wildlife seem to be injuries and, especially in the case of hedgehogs, surviving the winter. The background education and experience of respondents caring for different animal species varied, with respondents who only cared for hedgehogs having no animal-related education and limited experience of caring for wildlife.

In our study, the reported proportion of animals returned to the wild is very high, around 80%, compared to figures reported elsewhere of *circa* 40% in the UK (9) and Canada (3), and approximately 50% in the Czech Republic (1) and United States (17). As our respondents reported these figures for an example species they commonly cared for, our figures may not represent the whole picture. However, even considering that, the return rates are very high. The high rates raise our concern that the animals may have been returned to the wild in poorer conditions or admitted into care more lightly than elsewhere. Both options can lead to unnecessary suffering of the animals. The high recovery rates could, in principle, also be due to the good-quality care provided to wildlife casualties. However, it is unlikely that the quality of care provided in Finland would be significantly better than elsewhere, as very little regulations, guidance, education or control are in place. Release rates can also be expected to vary significantly among facilities based on the mixture of species being cared for and facility policies (5). However, the difference in numbers is so large that these reasons may not explain the whole difference.

Record keeping was uncommon among our respondents, as one in three respondents based their answers only on their own estimates and not even on partial records. This may distort the results, and it is possible that the high rates of animals returned to the wild are not real but rather reflect such rates that the respondents think would be desirable as we cannot rule out the effect of social desirability bias (18). The rehabilitators may think that very high release rates are a sign of success. We also observed that individuals without record keeping reported higher animal euthanasia rates before treatment and lower euthanasia rates during treatment than did respondents relying their estimates at least partly on record keeping. The estimated result may have biased our data towards over-estimating euthanasia at arrival and under-estimating it during treatment. Work assessment, preferably also including post-release monitoring, is essential for improving animal welfare (19). The lack of record keeping is worrying, as without it, rehabilitators have no way of monitoring their success or, for example, whether their treatment prognosis has been correct. This reduces the possibilities of learning and improving wild animal care. Further, post-release monitoring would be important to increase our knowledge of which animals survive and adapt back to their natural lives after release, as this is the only way to know whether rehabilitation is successful (2, 8, 9, 17). This is also the best way to estimate which practices are likely to yield the least total harm and to decrease the harm to animal welfare, human interests, and conservation (20). However, there are many constraints to post-release monitoring, with a lack of resources being a common one (19).

On the other hand, the international figures on release rates (1, 3, 9) are comparable to the figures reported by veterinarians in our study, as half of the wildlife casualties treated and reported by veterinarians were returned to the wild. Veterinarians also estimated the euthanasia rate at the time of animal arrival to be higher than did the other respondents in our data. This may be due to more severe cases on average being brought to veterinarians and veterinarians also having euthanasia methods easily available. Veterinarians are also used to using euthanasia in their daily work and have training to understand the negative consequences of further treatments to animals (6). We have previously reported from the same survey that veterinarians, regardless of the wildlife species they reported treating, were in many cases more likely to euthanize the animal than to initiate treatment (6). Overall release rates in our data were also much higher than the average 35 and 40% reported by SEY Animal Welfare Finland member associations for wildlife casualties in 2019 and 2022 (non-published data provided by SEY Animal Welfare Finland 2023). As is the case with many respondents in this study, SEY’s statistics are also, at least partly, based on estimates. We suggest that one reason for these differences in release rates may be that veterinarians and more organized NGOs incorporate more guidelines in their processes contrary to less-organized wildlife rehabilitators who may operate more according to their own principles. There may also be differences in insights to the stress and suffering the animal is experiencing due to different levels of training and experience.

High release rates may indicate that wild animals are being admitted on too light grounds. Some of these animals could potentially have survived even without treatment. Care of wildlife casualties can benefit animal welfare when performed appropriately (21). However, over-caring is a welfare problem, as treatments also causes multiple and cumulative stress to the animals, with negative consequences such as reduced resistance to disease, failed reproduction, and impaired cognitive ability, which can also lead to suffering and death of the seemingly healthy animal after release (13, 14, 22). Even when all else is well cared for, the animal is in good health and fit to be released, transport to a release site may have a significant negative effect on the animal’s state (14). Also, there are other possible negative consequences, such as the transmission of diseases and antimicrobial resistance, that should be considered (21). Preventing all unnecessary treatments would therefore be an important way to improve wild animal welfare (14, 17).

Another possibility is that the rates reported in our data indicate that animals have been released back into the wild in too poor condition, causing unnecessary suffering. Previously, volunteers have reported willingness to return animals back to the wild even with only little chance of survival (2, 6). When deciding which animals can be released back into the wild, the health of the animal is not the only issue to consider. The animal must also have appropriate skills to survive in the wild, such as finding food, seeking shelter, and avoiding predators. Additionally, the season should be suitable for release and appropriate habitat for the animal should be available, to name a few considerations (14). Rehabilitators have commonly been reported to lack criteria for assessing a suitable release site, and there are considerable differences in how they prepare their patients for release, if at all, along with how the animal’s condition is evaluated as suitable for release (2, 19). Additionally, there is a common lack of data on the survival of animals after release, and this under-resourced area also lacks good tools (2, 9, 13). Many factors affect the release rates of wildlife casualties, and until good follow-up systems are in place, release rates are not an adequate measure of the success of wild animal care. Instead, success could be measured, for example, by benchmarking the facilities and care programs against best practices and by keeping standardized records (5).

Less than 40 % of our respondents emphasized the need for further education on any specific issue of wildlife management. This is a surprisingly low proportion of respondents, given the wide range of skills required to manage wildlife and the fact that few managers are professionals. This may reflect an overestimation of skills and knowledge acquired and that gaps in knowledge are not identified, which unnecessarily increases the risk of compromising wild animal welfare while in care. Only one-fifth felt they required more knowledge to assess the ethical benefits and harms of treatment. This may be an underestimated need, as the release rates in our data suggest problems with animals being admitted into care on too light grounds or released in too poor condition. Many respondents felt they lack the knowledge needed to make a prognosis for the animal, and approximately one-third felt that they needed more skills to assess the condition of the animal to be returned to the wild. These skills are important both to prevent unnecessary treatments and to prevent the rehabilitation of animals not fit for life in the wild. In Finland, the rehabilitation sector mainly consists of volunteers, and there is not much legislation in place to regulate the work. Larger professional facilities are rare, which may impact wildlife care procedures. Training alternatives for wildlife care thus largely depend on the activity of individual rehabilitators. Caring for wildlife casualties does not require a permit nor specific training in Finland unlike in many other countries (1–3, 23, 24) although the law does require the carer of the animal to have sufficient skills to provide appropriate treatment (4). Based on our results, it should be considered that wildlife rehabilitation would be subject to licensing and mandatory training should be a prerequisite for obtaining a license. From January 2024 on, legislation will require that rehabilitators keep records and report their activities to the authorities (4). This will allow authorities to carry out animal welfare inspections of wildlife facilities to ensure that they are appropriate for the species kept and that adequate records are kept. This will also allow future research to be based on records rather than estimates.

Without proper standards and evidence-based science, rehabilitators’ decisions are likely influenced more by their own ethics and personal values (14). We and others have previously reported on the reluctance of rehabilitators without veterinary education to euthanize animals (2, 6, 7), even if the animal is not likely to survive in the wild (6). Some volunteers argue that any survival can be regarded as a successful release, even if most released animals do not survive, as some of them nevertheless do (2). This is, however, against the general recommendation that animals being released should have as good a chance of a similar survival span as their wild counterparts, otherwise rehabilitation should not be attempted (9, 15). This includes not only that the animal is fully recovered but also that it has all the skills required to survive (14).

Most respondents caring only for hedgehogs had no animal-related training and little experience in caring for wildlife, differing from respondents who also reported caring for other wildlife species in need. Although their activities were small in scale, and they only cared for a few hedgehog individuals at a time, the proportion of these respondents was high in our survey. The large number of respondents caring for hedgehogs may reflect the international trend showing an increase in hedgehog numbers admitted to rehabilitation in several countries in recent years (10, 11). The main reasons for admitting hedgehogs into care in Finland appeared to be similar to those in other countries, i.e., facilitating hedgehog hibernation and treating injuries and assumed orphans (10). Reports from other countries show release rates of around 40% (9, 10) for hedgehogs, which differs greatly from our results of approximately 90% in Finland. We found that hedgehogs were reportedly euthanized less frequently before or during treatment than other mammals were, which may indicate that hedgehogs are not captured from the wild in as poor a condition as other mammals and could also have been admitted into care on too light grounds, as has been reported elsewhere (10). The main reason hedgehogs were cared for was due to overwinter survival. We think it is important to consider less-intervening ways to help hedgehogs overwinter. As good nesting sites are a necessity for successful hibernation, providing these could be one option. Offering additional feeding in nature could help the animals gain enough body fat before hibernating, although feeding may also have adverse effects such as disturbing hibernation patterns (25). As hedgehogs are a protected species in Finland, it is illegal to capture them unless they are ill, injured, or otherwise in a helpless state (26).

Hedgehogs may also be returned to nature in too poor condition. Especially respondents who only cared for hedgehogs stressed that many rehabilitators are willing to rehabilitate hedgehogs even if the chances of returning the animal to the wild are small. As strictly hedgehog rehabilitators were all non-veterinarians, it may be affecting their reluctance to euthanize (2, 6). While it is possible for semi-fit hedgehogs to survive when provided with additional feeding and winter shelter in the vicinity of human settlements, risks are also associated, such as an animal’s ability to groom itself and therefore becoming infected with ticks, leading to welfare problems (9). However, although we observed differences between people who care for different wildlife species in our data, we cannot fully separate the influence of background training from the possible effect of the species being cared for.

Interestingly, people caring for large birds or birds of prey in Finland without veterinary training were more likely to consider suspected infectious diseases as a common reason for euthanasia than people caring for other species. On the other hand, bird rehabilitators were rather experienced; they manage several dozen large birds or birds of prey each year, with a median experience of approximately 10 years. We suspect their experience brought insight and confidence to remove a bird that is potentially dangerous to the wild population. As avian influenza is a serious disease (27, 28) that has been greatly discussed in the media in recent years, and cases of avian influenza has appeared in Finland regularly during last years (29), we suspect that it may also be linked to this result. Also, the Finnish Food Safety Authority (30) advises that a single bird that is found dead is considered avian influenza suspicion if the bird in question is a large bird of prey.

As our research is based on a freely available online survey, we cannot exclude the possibility of false information or multiple responses made by one person. Also, the number of people who care for hedgehogs, for example, may be disproportionately high because they are very organized in social media and thus easy to reach. However, as no official list of wildlife rehabilitators was known at the time of our survey, we considered an open survey was the best way to reach also those rehabilitators that do not work in larger establishments or NGOs. We felt that this gave us a broader perspective on the situation in the country. We minimized the risk of inappropriate responses by explaining the purpose of the questionnaire and by designing the questionnaire such that participation in the survey required some focus and effort on the part of the respondent.

## 5 Conclusion

These findings raise concerns of unnecessary welfare problems regarding wildlife casualties due to animals either being admitted on too light grounds or returned to the wild in too poor conditions. Proper record keeping should be maintained to help estimate whether wildlife care is targeted at individuals in need, that the care is well designed, and that animals are released in good condition. Options for post-release monitoring should be sought. More research is needed to understand what the high release rates found in our research reflect or if the lacking records even reflect the reality. The balance between survival chances of the animals in the wild, euthanasia, and potential harm due to human care is a delicate question and careful ethical consideration is necessary. These results indicate that both the education and experience of the rehabilitators, and the animal species being cared for may affect decision-making. Thus, more education should be made available and even mandatory.

## Data Availability

The raw data supporting the conclusions of this article will be made available by the authors, without undue reservation.
