# Supplementary material for: Reasons for admission and rehabilitation rates of various wildlife species in Finland
Source: Front Vet Sci. 2024 Oct 2;11:1455632. doi: 10.3389/fvets.2024.1455632 (PMC11479885; doi:10.3389/fvets.2024.1455632)
Supplement: Supplementary file 1 [file Data_Sheet_1.pdf]

Supplement 1. A list of questions reported here

Gender
Woman
Man
Other
I don’t want to answer

What year were you born?

Your latest education?
Primary School
Vocational School or Upper Secondary School
University or University of Applied Sciences

Are you a veterinarian?
Yes
No

Do you have some other animal related education?
Yes
No
Other, what?

What is your latest animal related education?
Vet nurse
Farmers substitute worker (ie. educated farm relief worker)
Other, what?

How long (in years) have you been rehabilitating wild animals?

Even though the years might differ from each other, think about a regular year in your activities. Estimate, how many individual animals belonging to each group given below do you care for in a year on average. Please, mark as numbers.
mammals
birds
reptiles
amphibians
fish

List the most typical animal species that you care for on each group of animals
mammals
birds

reptiles
amphibians
fish

Which of the following are the most common reasons why wild animals come into your care each year? You may choose multiple answers.

Orphans or animals thought to be orphans.
Injured animals.
Sick animals. ´
Animals that due to their condition may not survive overwinter in the wild.
Animals that do not behave normally. ´
Something else, what? ´

Next, think about species that you most typically care for and estimate the survival of these species during or after treatment and the final placement of the animal.
Species:

How large percentage of the individual animals of the species you wrote above
Is returned to the wild:
Is placed in a zoo:
Has to be euthanized during care:
Has to be euthanized before care:
Dies in your care:
Total:

Was your answer to the questions above based on
Your records
Your estimate
Both

Think about a typical case in which you ponder euthanasia of an individual animal. Estimate, how much do you agree or disagree with the statements below. (1 = fully disagree, 7 = fully agree):

I don't as easily euthanize endangered species, but I try to treat them longer than less endangered species.
If the costs of the treatment are starting to become high, I end up euthanizing the animal.
I end up euthanizing animals brought to my care because there is no veterinary help available for them.
I end up with euthanasia if I evaluate that the treatment will cause the animal a lot of stress.
Whether the animal will survive in nature or not after treatment does not have any impact on my decision to euthanize.
I end up with euthanasia if I evaluate that the treatment will cause the animal a lot of pain.

It is unlikely that I euthanize the animal if it can be easily returned to live in the wild.
I end up with euthanasia if the ailment of the animal requires more than half a year of treatment and the animal isn't of a species that hibernates.
I end up with euthanasia if the animal may spread contagious diseases to the wild.
I end up with euthanasia if the ailment of the animal requires more than half a year of treatment of which the animal would hibernate part of the time.
Something else, what?

How much do you agree or disagree with the following statements (1=fully disagree, 7=fully agree): If there is no known end-of-life solution for the animal at the moment it is brought to me (rehabilitating back to the wild, a place in an enclosure for wild animals)

the animal should in my opinion be euthanized immediately regardless of the species and of its condition.
I could start treating it whatever its species and condition, but any end-of-life solution should be known within two days at most.
whether or not it is of an endangered species, treatment can start while an end-of-life solution is being sought.
and it is of an endangered species, it can be treated indefinitely until an end-of-life solution is found.
it should be euthanized immediately if the species in question is common.
I will start the treatment but only if it is unlikely to stress the animal very much.
I would start treatment only if it was unlikely to cause a lot of pain and suffering.
Something else, what?

How much do you agree or disagree with the following statements (1=fully disagree, 7=fully agree): I think a wild animal

Should be rehabilitated only if it is very likely to be returned to the wild.
Should be rehabilitated if it is quite likely to be returned to the wild.
Should be rehabilitated if there is even a small chance to be returned to the wild.
Should be rehabilitated if there is a place for it in a zoo.
Should be rehabilitated even if the animal would be partly dependent on humans for the rest of its life
(requires additional feeding, but otherwise lives in the wild).
Should be rehabilitated even if the animal would be fully dependent on humans for the rest of its life (fully tame, doesn't seek species companion, doesn't know how to find food, lives under human influence).

Think about wild animal species and the different parts of care they need. In which issues do you think you most need more knowledge. You may give a list of species under each statement or give your answer generally speaking.
Estimating the animal's need for care
Estimating the prognosis (

Estimating the harm and benefit relating to the care of the animal
Proper feeding and watering of the animal.
Proper other basic non-medical care of the animal.
Medical care of the animal (list procedures and species)
Estimating the fitness of the animal for returning it to the wild.
Returning the animal back to wild in practice.
Teaching a young animal species specific skills so it can survive in the wild.
Something else?
